# Expanding horizons in complement drug discovery: challenges and emerging strategies

**DOI:** 10.1007/s00281-017-0655-8

**Published:** 2017-10-06

**Authors:** Claire L. Harris

**Affiliations:** Complement Therapeutics Research Group and National Renal Complement Therapeutics Centre, Institute of Cellular Medicine, Newcastle University, 3rd floor William Leech Building, The Medical School, Framlington Place, Newcastle upon Tyne, NE2 4HH UK

**Keywords:** Complement, Drug discovery, Therapeutics, Eculizumab, Disease, Inflammation

## Abstract

The complement system is best known for its role in innate immunity, providing a first line of defence against infection, maintaining tissue homeostasis by flagging apoptotic cells and debris for removal, and orchestrating crosstalk between adaptive and innate immunity. In a growing number of diseases, complement is known to drive pathogenesis or to contribute as an inflammatory amplifier of a disease trigger. Association of complement with common and devastating diseases has driven an upsurge in complement drug discovery, but despite a wealth of knowledge in the complexities of the cascade, and many decades of effort, very few drugs have progressed to late-stage clinical studies. The reasons for this are becoming clear with difficulties including high target concentration and turnover, lack of clarity around disease mechanism and unwanted side effects. Lessons learnt from drugs which are either approved, or are currently in late-stage development, or have failed and dropped off the drug development landscape, have been invaluable to drive a new generation of innovative drugs which are progressing through clinical development. In this review, the challenges associated with complement drug discovery are discussed and the current drug development landscape is reviewed. The latest approaches to improve drug characteristics are explored and those agents which employ these technologies to improve accessibility to patients are highlighted.

The complement system is best known for its role in innate immunity, providing a first line of defence against infection, facilitating phagocytic uptake of pathogens and in some cases directly killing pathogens or infected cells through lysis. The highly inflammatory mediators generated as a by-product of activation play a crucial part in signalling to surrounding cells and to migrating leukocytes that there is danger in the environment [1, 2]. Complement also plays a key role in tissue homeostasis, flagging apoptotic cells and debris for removal, guiding immune complexes to the reticuloendothelial system for clearance, and orchestrating crosstalk between adaptive and innate immunity. Unfortunately, complement contributes to pathogenesis in a number of diseases; in some cases, it drives pathology, in others it amplifies or exacerbates the inflammatory and damaging impact of non-complement disease triggers. Despite a wealth of knowledge in the complexities of the complement cascade, and many decades of endeavour, very few drugs have progressed in clinical studies. Recently, strong genetic associations of complement with common diseases have emerged and fuelled the fire of complement drug discovery leading to an explosion in complement therapies in development; whilst many of these agents and others before them have failed to progress, their legacy is key to future success. The drug development landscape is now littered with dead or dying assets that perfectly exemplify the challenges associated with ‘drugging’ the complement system. This review will briefly scan the current drug development landscape and focus on the challenges of complement drug discovery and lessons learnt from past and present clinical studies. Innovative approaches that are emerging to overcome obstacles blocking success will be explored, highlighting drugs in development which employ these state-of-the-art strategies.

## Complement activation and control

Complement is activated by various different mechanisms, and all converge at the point of C3 cleavage but differ according to the nature of activation. The classical pathway is triggered by a ‘lethal array’ of antibody on a target surface, the lectin pathway is initiated when a lectin such as mannan-binding lectin (MBL) binds a pathogen, and the alternative pathway constantly ‘ticks over’ in fluids, priming the system to enable rapid response in the face of infection; this background turnover of complement results in continual surveillance of tissues and preservation of health; there are many excellent reviews in this area [1–4]. It is also clear that complement links to adaptive immunity by engaging receptors on immune cells, such as B cells, and supporting the generation of an effective immune response. Opsonisation of antigens with the complement fragment, C3dg, results in co-engagement of the B cell receptor and complement receptor 2 (CR2; CD21) and synergistic downstream signalling, lowering the threshold concentration of antigen required to activate the cell [5, 6]. Multiple complement activation fragments (C3a, C3b, C5a) bind respective receptors and influence T cell differentiation and maturation of antigen-presenting cells depending on the surrounding cytokine milieu; these effects can be very localised and driven by complement synthesised in specific tissues [7].

**Fig. 1:**
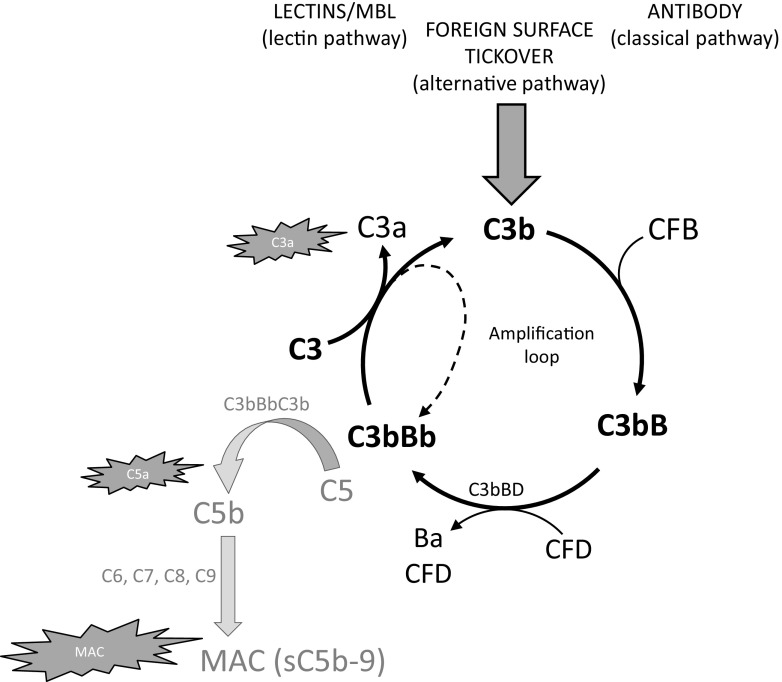
The complement amplification loop. Complement is activated by various triggers including binding of lectins or antibody to a target surface or by background ‘tickover’ of the alternative pathway. The pathways converge at the point of C3 cleavage to C3b and C3a by the C3 convertase (either C3bBb or C4b2a). The larger cleavage fragment, C3b, binds CFB to form a proenzyme which is activated by CFD to form further convertase, C3bBb. This enzyme cleaves many molecules of C3 to C3b each of which is capable of amplifying the pathway as illustrated in the cycle (in bold), or forming the C5 convertase, C3bBbC3b (grey), which cleaves C5 to C5b and C5a and triggers the terminal pathway. This feedback cycle amplifies the signal from any small trigger to a large downstream effect. The proinflammatory by-products of complement activation are the anaphlylactic and chemotactic peptides, C3a and C5a and the membrane-associated macromolecular complex, MAC

Complement activates in an exponential way due to an internal amplification loop [8]. The largest fragment of activated C3, C3b, produced due to the actions of any of the activating pathways, can bind factor B (CFB) to form the proenzyme C3bB. Binding of factor D (CFD) results in proteolytic cleavage of CFB and formation of the C3 convertase, C3bBb. This is a labile enzyme with a half-life of only minutes due to irreversible dissociation of components, but binding of properdin can stabilise and prolong activity for up to half an hour. During its lifetime, the C3 convertase cleaves many molecules of C3 to C3b, each with potential to form a C3 convertase in its own right. This results in an amplifying cycle right at the heart of the cascade which transforms a small trigger to massive downstream effect (Fig. 1).

Unfortunately, this volatile and rapid-acting system can do harm as well as good. In health, the system is constantly activating, but that is in perfect balance with control mechanisms which allow sufficient flux through the pathway to recognise appropriate (foreign) targets, but limit ‘tickover’ amplification and activation on self-surfaces [3, 9]. There are various control mechanisms which inhibit the cascade:

i) Inherent instability; examples include the short half-lives of complexes such as C3bBb, and the limited time for which nascent C3b can bind covalently to a target, or that C5b can bind C6 to form C5b6.
ii) Decay accelerating activity; various proteins on cell surfaces (CD55, CD46, CD35) and in fluids (complement factor H (CFH), C4b-binding protein) bind to the C3 and C5 convertases and accelerate the labile decay of the components resulting in virtually instantaneous loss of the proteolytic subunits (such as Bb).
iii) Cofactor activity; this is inherent to CD46, CD35, CFH and C4bp; binding to their ligand (C3b, C4b) enables a soluble serine protease, factor I (CFI), to bind the complex and proteolyse either component preventing further proenzyme and convertase formation.
iv) Inhibition of the lytic pore, the membrane attack complex (MAC); the widely expressed MAC inhibitor, CD59, binds to the terminal proteins as they start to form the MAC and prevents polymerisation of C9. Other proteins present in the fluid phase, S-protein, clusterin and even C8, bind to the activated MAC components before they have a chance to associate with a membrane and render the complex soluble.

## Complement and disease

**Fig. 2:**
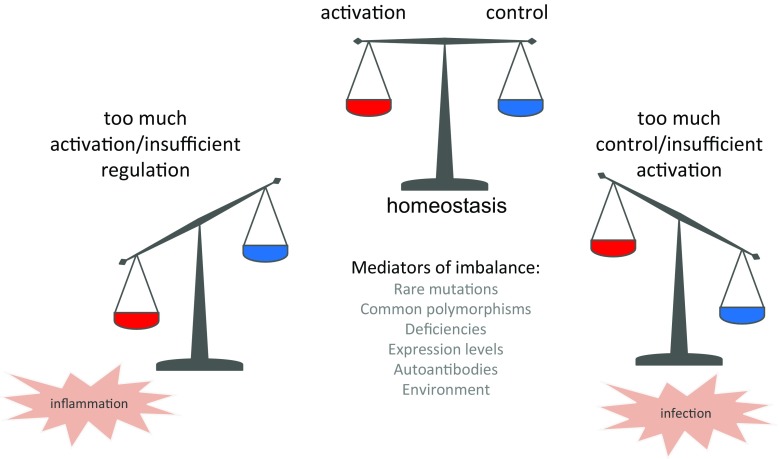
Complement activation and control and mechanisms of dysregulation. The complement system is always activating at a background ‘tickover’ level which enables rapid response in the face of infection; activated C3b deposited on a non-self (or unprotected) surface will fire the amplification loop. In order to prevent harm to self-tissues, an armoury of complement regulatory or control proteins exists in plasma and on self-cells to inactivate complement as it becomes activated, these proteins include factor H (CFH), CD46, CD55 and CD59. In health, there is sufficient flux through the system to drive activation on foreign cells, whilst damage to self is prevented. If this perfect balance between activation and control is disturbed, then tissue damage and disease may ensue. Various mechanisms tip the balance in favour of inflammation or infection and those are illustrated here. These include mutations and polymorphisms which affect function of the activating or control proteins, expression levels of these proteins (which may in some cases be zero), autoantibodies which drive complement in an inappropriate manner, and environmental triggers

Various mechanisms can disrupt this balance resulting in over-activation, damage and disease (Fig. 2); there are already many outstanding reviews in this area [10–15]; disease triggers may be inherited or acquired and are only described in brief here. Mutations in the complement genes translate to proteins which can cause loss or gain of function. Loss of function of an inhibitory protein, such as CFH which controls the amplification loop, can result in over-activation of the system and inflammation. Similarly gain-of-function changes in activating proteins can cause imbalance, often impacting in a similar way by weakening affinity of control proteins for their ligand (binding of CFH to its major ligand, C3b, or to self-surfaces where it restricts activation) [13]. Common changes in complement proteins (polymorphisms) can also impact function, although the effect on function is often slight and only apparent when risk polymorphisms occur concurrently in multiple proteins [16]. Expression levels of activating components and regulators vary enormously in the healthy population, but drastic losses in expression, due to a non-sense mutation, for example, can upset the balance and cause loss of control. This mechanism is evident in diseases such as age-related macular degeneration (AMD) where low level expression of the enzyme CFI results in inefficient conversion of C3b to the downstream fragment, iC3b [17]. Acquired drivers of disease include autoantibodies, for example to the acetylcholine receptor in myasthenia gravis, which can drive complement activation in an inappropriate manner. Autoantibodies can bind to components and complexes and alter function; for example, nephritic factors can stabilise the C3 and C5 convertases and are associated with diseases such as C3 glomerulopathy (C3G) and acquired partial lipodystrophy (APL). Environmental triggers also play a role and an example of this might be oxidation of lipids in the retina of individuals at risk of AMD, or pregnancy in the case of individuals at risk for atypical haemolytic uremic syndrome (aHUS) [18].

The complement cascade is an excellent example of a biological pathway whose activation and progression is characterised by an exquisite set of conformational transitions [19–21]. A conformational change in one activating protein reveals binding sites for the next in the cascade, which in turn changes shape upon binding and facilitates engagement of the following component. Early steps in the pathway are characterised by proteolytic cleavage (for example, C3 to C3b, CFB to Bb, C5 to C5b), whereas later stages of the pathway simply involve conformational transitions subsequent to a binding interaction (C6 through to C9 and MAC formation), Fig. 1. Given the plethora of diseases in which complement is activated, the cascade presents an enticing challenge for drug discovery with potential to inhibit enzymes, prevent protein/protein interactions and interfere with conformational transitions.

## The current drug development landscape

Complement drug discovery is not a new concept; recombinant DNA technology has long been used to generate soluble forms of complement control proteins to bolster circulating levels of inhibitory proteins. These agents have had success in animal models of disease, and have even made it in to man (for example, TP10; soluble complement receptor 1, sCR1), but have so far struggled to make it to, or to progress through, clinical development [22, 23]. An exception is C1 inhibitor (C1inh) which is used to treat hereditary angioedema (HAE); HAE is triggered by a lack of C1inh which controls the contact-system protease, kallikrein, as wells as the activating complex of the classical pathway, C1; however, C1inh, either recombinant (Ruconest) or native (Cinryze, Berinert), is used to replace missing protein, rather than to block complement as a therapeutic target [24]. Small molecule inhibitors of the complement cascade have been sought for several decades but often show off-target effects and toxicity in animals. Only recently have various small molecule inhibitors of the enzymes CFB and CFD been developed and translated to man; these are currently undergoing early-stage clinical trials [25, 26]. Antagonism of complement receptors by small molecules has also been tried and tested over many years with limited progression to man. One such inhibitor of the C5a receptor (C5aR1), termed avacopan or CCX168, is now reaching the late stages of clinical development; it is approaching phase 3 studies for ANCA vasculitis [27].

**Fig. 3:**
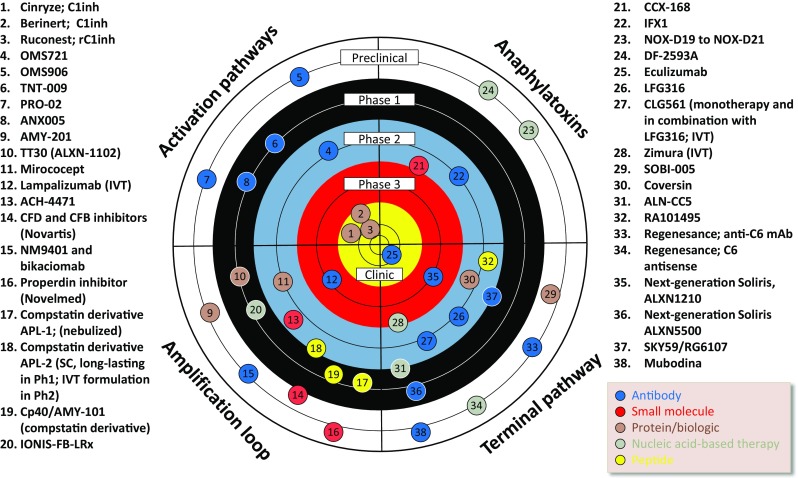
The current complement drug development landscape. This schematic illustrates drug which are being developed for clinical use, either approved (bullseye, yellow) or in the pipeline (remainder of target; clinical trial, black-red; preclinical, white). Complement drug discovery is a rapidly moving field and, inevitably, some compounds will progress, others will fail and new drugs will emerge to take their positions in the landscape in the coming years. Antibodies (whole Ig or fragments) in development or approved include eculizumab (Alexion), a blockbuster drug approved for treatment of PNH and aHUS, and its follow-up molecule ‘next-generation eculizumab’ ALXN1210 (phase 3). Lampalizumab (Genentech/Roche) is in phase 3 for geographic atrophy, IFX-1 (InflaRx) is an anti-neo C5a, and LFG316 and CLG561 are specific for C5 and properdin (both developed by Novartis). Other antibodies which are pathway-specific include OMS721 and OMS906 (Omeros) against lectin pathway proteins, TNT009 (True North therapeutics) specific for C1s, PRO-02 (Prothix) against C2, and ANX005 (Annexon Biosciences) against C1q. Novelmed have antibodies against CFB (bikaciomab) and properdin (NM9401) in preclinical development. Other antibodies block C5 or MAC and include SKY59 (Chugai/Roche)—a sweeping antibody against C5, ALXN5500 (Alexion) specific for C5, Regenemab—an antibody specific for C6 (Regenesance) and Mubodina (Adienne Pharma & Biotech), an antibody against C5 being developed for typical HUS. Cinryze, Berinert and Ruconest are all C1 inhibitor, either native (Cinryze, Berinert) or recombinant (Ruconest)—approved for HAE. Various other biologics target the alternative pathway including TT30 (Alexion), Mirococept (King’s College London/MRC) and AMY-201 (Amyndas). SOBI-005 (SOBI) is an affibody and Coversin is a recombinant tick protein, both biologics which block C5. Peptide-based agents include the alternative pathway blockers, all derived from Compstatin: AMY-101 (Amyndas), APL-1 and APL-2 (both Apellis Pharmaceuticals), and the C5-blocking peptide from RaPharma, RA101495. Small molecules which target complement have been slow to develop but success is now evident with CCX-168 (ChemoCentryx; C5a-receptor blocker) and ACH-4471 (CFD blocker, Achillion Pharmaceuticals); trials of the SM inhibitors of both CFD and CFB (Novartis) are anticipated. Small molecule inhibitors of properdin (Novelmed) have been described although recent development is not reported. There are a small number of nucleic acid-based therapies which have been developed which bind target and block function, these include Zimura (Ophthotech), an aptamer which blocks C5, NOX-D19-D21 which bind C5a (Noxxon Pharmaceuticals) and DF-2593A (Dompé) which binds C5a receptor and blocks via an allosteric mechanism. Finally, various agents which function at the gene expression level are under development and these include antisense for CFB (IONIS-FB-LRx; Ionis Pharmaceuticals), RNAi for C5 (ALN-CC5, Alnylam Pharmaceuticals), and an agent under development by Regenesance based on locked nucleic acid (LNA) technology, which prevents C6 expression. This figure is adapted from Morgan BP, Harris CL. Nat Rev. Drug Discov. 2015 Dec;14(12):857-77; ‘Complement, a target for therapy in inflammatory and degenerative diseases’ [30]

Complement knockout animals have illustrated the potential for blockade of components for therapy in animal models; similarly, blocking antibodies have shown promise, but limitations around target concentration and local biosynthesis are clearly evident in models and in man. Various strategies to block or ‘knockout’ components in man have been tested in the clinic, these include monoclonal antibodies against complement components or nucleotide-based therapies (antisense, RNAi) which primarily target the liver, the main (but not only!) source of complement in man [28]. One agent, a humanised monoclonal against C5, eculizumab, is the first-in-class drug approved for two orphan diseases, paroxysmal nocturnal hemoglobinuria (PNH) and aHUS [29, 30]. Various other strategies have been tried and tested, with varying success (Fig. 3).

The drug development landscape is littered with agents that have failed at the preclinical or early clinical stage [30]. Their modes of action and modalities are wide-ranging. It is becoming increasingly clear that an understanding of disease mechanism and matching of drug modality and mode of action to the right disease and patient population (or stratified subpopulation) is critical to success. There are some disease indications where studies of genetics, biomarkers and tissues illustrate a primary role for complement in pathogenesis (for example, AMD, aHUS, C3G, PNH) and others where the primary trigger is not complement but complement acts to exacerbate downstream inflammation and tissue damage (for example, rheumatoid arthritis, systemic lupus erythematosus). A range of different drugs, or combination of drugs, will be needed for effective management of the many and diverse complement-mediated diseases. This figure is adapted from Morgan BP, Harris CL. Nat Rev Drug Discov. 2015 Dec;14(12):857–77; ‘Complement, a target for therapy in inflammatory and degenerative diseases’ [30].

## Challenges in anticomplement drug development

### Disease mechanism

The reasons behind the low success rate for anticomplement drugs are clear. An in depth understanding of disease mechanism and the contribution of complement is critical to success and that knowledge has not always been there. This understanding will include knowledge of the disease trigger(s) and its link(s) to the complement activation pathway(s) involved (lectin, alternative, classical and terminal). Complement activation fragments deposited in diseased tissue or circulating in blood at elevated levels, indicate complement activation in pathology, but do not necessarily pinpoint a primary role in disease, or distinguish cause from effect. Not only is knowledge of the causal pathway extremely important to target the right therapy to a disease indication but homing in on just one arm of the system leaves other pathways to function and preserve the infection-fighting and life-preserving properties of complement. Examples of drugs in clinical development which target a specific pathway include TNT009 (True North Therapeutics; acquired by Bioverativ in 2017) which is a blocking antibody against C1s, an enzyme specific to classical pathway. This is being tested in antibody-driven diseases such as bullous pemphigoid and cold agglutinin disease. Omeros is developing a monoclonal antibody (OMS721) against the lectin pathway-specific enzyme, MASP-2, for thrombotic miroangiopathy and various renal indications. Perhaps the best known agent is eculizumab, an antibody against C5 which selectively inhibits C5 activation and MAC formation and leaves the activation pathways and therefore the pathogen-opsonising functions of complement unaffected [29].

Knowledge of disease mechanism is built using a variety of data. Animal models can provide powerful insight, although care must be taken when extrapolating results to man [31]. Genome-wide association studies (GWAS) and candidate gene studies have provided powerful data over the last decade, shedding light on complement associations with common and devastating diseases such as AMD and Alzheimer’s disease (AD) [15, 32]. Genetic association of multiple proteins within a biological pathway have been particularly enlightening. Association of multiple proteins in the amplification loop, both activating and controlling proteins, with AMD provide powerful evidence for this pathway as a primary mediator of pathology [16]. Proteins associated with risk include the activating proteins C3 and CFB and the control proteins CFH, CFI and the CFH-related proteins (CFHRs); indeed, intravitreal inhibition of the amplification loop with an inhibitor of CFD (lamaplizumab) is a strategy which showed promise in phase 2 studies of geographic atrophy and is currently being tested in phase 3 trials [33]. Development of this agent was likely driven by the genetic studies which highlighted the amplification loop as a primary target in this disease. Common polymorphisms alter risk for disease, but alone, do not cause disease. Advances in genetic screening mean that strategies such as whole exome sequencing and multiplex ligation-dependent probe amplification (MLPA) are affordable and rapid and are now commonly used to identify mutations and genetic changes in individual patients which are highly penetrant and are disease-causal on their own. Very often, these genetic analyses are used in the clinic for diagnosis, but ability in this current era to move from patient phenotype, to causal gene and then to functional dissection of pathogenic mechanism in the research environment is incredibly powerful and provides unprecedented insight into disease mechanisms [13].

Matching the drug to mechanism is one key to success. Stratifying within a patient population might also be important. For example, C3G is a disease caused by dysregulation of the amplification loop which causes renal failure. Therapy with the anti-C5 mAb eculizumab has been hit and miss, with some patients showing improvement and others none. It is becoming increasingly clear that some individuals within that patient population show distinct dysregulation at the level of the C5 convertase which may be a driver to an acute inflammatory phenotype within the kidney; it is entirely possible that these patients, *and these alone*, might benefit from anti-C5 therapy [34, 35]. In the UK, the National Health Service England (NHSE) has approved use of eculizumab only for those patients who have confirmed activation of C5 and deposition of MAC within a transplanted kidney [36]. Stratification of patient populations is probably critical for successful outcome of clinical trials and goes beyond C3G to include disease indications such as myasthenia gravis (positivity for complement-activating anti-ACHR antibodies) and AMD (presence of a risk genetic makeup or ‘complotype’). In the MAHALO phase 2 trial (Genentech) in geographic atrophy, the CFD inhibitor, lampalizumab, slowed disease progression most prominently in individuals with a particular genetic makeup which notably linked to a polymorphism in the CFI gene [33]. It is noteworthy that a recent press release from Genentech (September 8, 2017) reported that the phase 3 study (SPECTRI) did not meet its primary endpoint of reducing mean change in geographic atrophy lesion area; it is not yet clear whether genetic analyses have been performed.

### Side effects of therapy

Beyond identifying disease mechanism, there are clear hurdles to overcome in the drug development process, including side effects of inhibiting the complement system, such as risk of infection [2, 37]. An inability to form MAC is associated with increased risk of infection with the Gram-negative bacterium *Neisseria meningitides*, as illustrated in individuals with genetic deficiency of a terminal pathway protein such as C6. Deficiency of a protein earlier in the cascade, such as C3, CFB or CFD is associated with a wider range of infections as ability to opsonise with (i)C3b is also impacted. The number of families harbouring a (homozygous) genetic defect in these proteins are limited, but studies indicate that individuals suffer from recurrent bacterial infections with diverse organisms, including of the genera *Neisseria meningitidis*, *Enterobacter aerogenes*, *Haemophilus influenzae*, *Escherichia coli*, *group A streptococcus*, *Streptococcus pneumoniae*, *Streptococcus pyogenes* and *Staphylococcus aureus* [38]. There are a number of strategies employed to mitigate these infection risks, these include prophylactic antibiotics and vaccination. Vaccination against *Neisseria meningitides* is a prerequisite prior to administering eculizumab, whereas multiple vaccinations may be required to overcome risk encountered by inhibiting the amplification loop. Apellis Pharmaceuticals are in early clinical trials in PNH of an agent, APL-2, developed from the C3 inhibitory peptide, compstatin [39]. Trial inclusion criteria include documented evidence of administering *Neisseria meningitides* vaccine, Pneumococcal conjugate vaccine (multivalent) or Pneumococcal polysaccharide vaccine 23 (PCV13 or PPSV23) and *Haemophilus influenzae* Type B (Hib) vaccine (www.clinicaltrials.gov; NCT02588833). Various forms of compstatin (APL-2, AMY-101 (Amyndas)) are currently in the clinic with agent being administered systemically; the presence or absence of adverse events in these trials will be incredibly informative to the drug discovery field. Intriguingly, an interim report on the Apellis phase 1b studies (NCT02588833, NCT02264639) was recently released by the company (press release, June 29th 2017). It indicated that there were no significant drug-related safety concerns over the 6-month systemic treatment with APL2, further data are anticipated.

Achillion Pharmaceuticals and Novartis are both progressing small molecule inhibitors of CFB and CFD and are pioneering studies of relative infection risk due to inhibition at either the C5 level or within the amplification loop. In vitro studies using whole blood have been reported and indicate a greater negative impact on bacterial clearance from C5 inhibition as opposed to CFB/CFD [40]. In these assays, the chemotactic/anaphylactic peptide, C5a, is clearly required for effective activation of leukocytes in order to engulf pathogens. When the amplification loop is inhibited, the classical and lectin pathways in an adult may be sufficient to provide adequate C5a for cell recruitment and activation. It remains to be seen whether the augmentative effects on adaptive immunity of the C3 and C5 activation fragments are impacted following long-term administration of these inhibitors. It could be speculated that blockade of C3 and C5 may impact the efficiency of vaccination, particularly with respect to activation of memory cells [7].

Complement also has key roles in tissue homeostasis and preserves health by facilitating removal of immune complexes, apoptotic cells and debris; thus, interfering with these clearance mechanisms could pose a risk to health. As an example, individuals with a genetic defect in the classical pathway have high risk of developing lupus-like symptoms; 93% of individuals lacking C1q and 57% lacking C1r/s present with a lupus-like illness [41]. It is not known whether risk will be similar in individuals who acquire deficiency later in life due to therapeutic inhibition, but current clinical trials, such as that run by True North Therapeutics (phase 1, NCT02502903; anti-C1s antibody), may shed light on this issue. Participants are vaccinated against *Neisseria meningitidis*, *Haemophilus influenzae* and *Streptococcus pneumoniae* to mitigate infection risk, and clinical biomarkers will inform on the safety profile.

Infection risk can also be mitigated by selective targeting of the complement pathway. Although a defect may be present in activation pathways, if the pathogenic mediator is downstream, then the drug target does not necessarily need to be within the defective pathway. Examples of this are PNH and aHUS. In both diseases, blockade of C5 with eculizumab results in profound clinic benefit, yet in both indications there is a clear defect in the activation pathways. Eculizumab prevents the proinflammatory impacts of C5a and MAC evident in aHUS, and prevents the MAC from lysing and activating cells in PNH; thus, blockade at the level of C5 can relieve symptoms [42]. In both these treatment regimes, vaccination is only required against those pathogens that are directly lysed by MAC (*Neisseria meningitidis*). By inhibiting downstream of the defective pathway, the opsonising properties of the activation pathways and amplification loop are preserved. However, there may be drawbacks to this strategy and this is evident in the eculizumab-treated PNH population. A large number of patients are rescued from transfusion-dependent status due to anti-C5 therapy, but it is becoming increasingly clear that therapy triggers a secondary clinical complication in PNH [43]. Defective erythrocytes in untreated individuals normally lyse due to MAC formation, but when MAC is blocked these cells become highly decorated in fragments of activated C3 due to lack of the GPI-anchored inhibitor of the amplification loop, CD55. A subset of individuals remain transfusion-dependent due to extravascular (rather than intra-vascular) hemolysis of these C3 fragment-labelled cells. If a drug does not rectify the original disease-triggering problem, it is clearly important to monitor any magnified secondary pathology due to that ongoing causative defect.

## Target concentration and turnover

Risk to health posed by therapeutic inhibition of complement is one challenge facing complement drug discovery and development. Another obstacle in complement drug discovery is target concentration. Unlike most cytokines which are present (transiently) in plasma at just picograms per millilitre, complement proteins are present at huge amounts; between 3 and 5% of total plasma protein are complement proteins! Complement proteins are released by the liver and also at many extrahepatic sites [28]; in inflammatory disease, local biosynthesis can contribute significantly to complement in that environment. Target concentration and turnover both impact on drug dose—it is understandable why eculizumab has to be administered intravenously at 1200 mg every other week to patients being treated for aHUS as levels of the target, C5, are high at between 90 and 172 mg/litre in the healthy population (0.44 μM average concentration; reference ranges according to Cardiff and Vale University Health Board Immunology Reference Laboratory; http://www.cardiffandvaleuhb.wales.nhs.uk/immunology); even at high doses, breakthrough can occur [44]. Requirement for high dose of antibody not only costs more but also limits ability to administer drugs via a subcutaneous route, which would be ideal for long-term therapy; a single subcutaneous injection is limited to 1–2 ml volume of antibody at between 100 and 150 mg/ml (approximately 2–3 mg/kg in human). Additional problems may be encountered when an individual is ill, as most complement proteins are acute phase reactants and thus levels can increase and breakthrough symptoms may occur due to insufficient blockade. There are some complement proteins which are synthesised only at extrahepatic sites, such as C1q, C7 and properdin, and these may therefore present as better targets. However, levels are not necessarily low (C7 normal range is 55–85 mg/litre) and local biosynthesis at sites of inflammation and disease, rather than systemic levels, may have profound effect on pathogenesis.

The impact of turnover on drug dose is illustrated by agents which target the amplification loop protein, CFD. CFD is a small protein subjected to high renal filtration; it is reported that CFD turnover might be as high as 1.33 mg/kg/day [45, 46]. In vivo studies in cynomolgus monkeys with lampalizumab, a humanised Fab which binds the CFD exosite with picomolar affinity, indicated that the pharmacokinetic/pharmacodynamic (PK/PD) properties of this agent were unfavourable when administered systemically [47]. High doses were required to block the amplification loop and concentration of CFD increased tenfold in plasma due to antibody-mediated antigen accumulation. This agent is given locally into the eye in humans and is in phase 3 for geographic atrophy; however, intravitreal therapy clearly has limitations for a large patient population, particularly if early-stage (pre-blinding) disease is to be targeted and development of late-stage disease prevented. It remains to be seen whether systemic inhibition of the complement system has any impact on progression of AMD. Another asset which inhibits CFD, ACH-4471 (Achillion Pharmaceuticals), can be given via oral dosing—an exciting breakthrough for the field. Interim data from the phase 1 study indicated that the dosing was high, and administration was required several times a day to totally suppress the amplification loop [48]. Nonetheless, the data indicate that oral medication can suppress the complement system. It remains to be seen how this agent, and others like it, will fare in a disease state.

Whilst it is clear that delivery issues are a major impediment to effective anticomplement therapy, the field has advanced rapidly in the last decade with a plethora of clinical studies progressing; we are in a place where hard evidence from failed and successful studies in man has already, or will in the near future, back up or refute concerns around dosing or side effects. Data from clinical studies have galvanised and guided drug development and we see a new generation of enhanced drugs emerging in the clinic; for example, next-generation eculizumab (ALXN1210) is a version of the original drug engineered to enhance its PK properties (see below). It is in phase 3 studies and clinical data demonstrate that this anti-C5 antibody can be dosed every other month, rather than every other week. Innovative strategies such as those described below to decrease dose and enhance effective delivery are emerging and will dominate the complement drug landscape in the next decade.

## Engineered antibody strategies to reduce dose

**Fig. 4:**
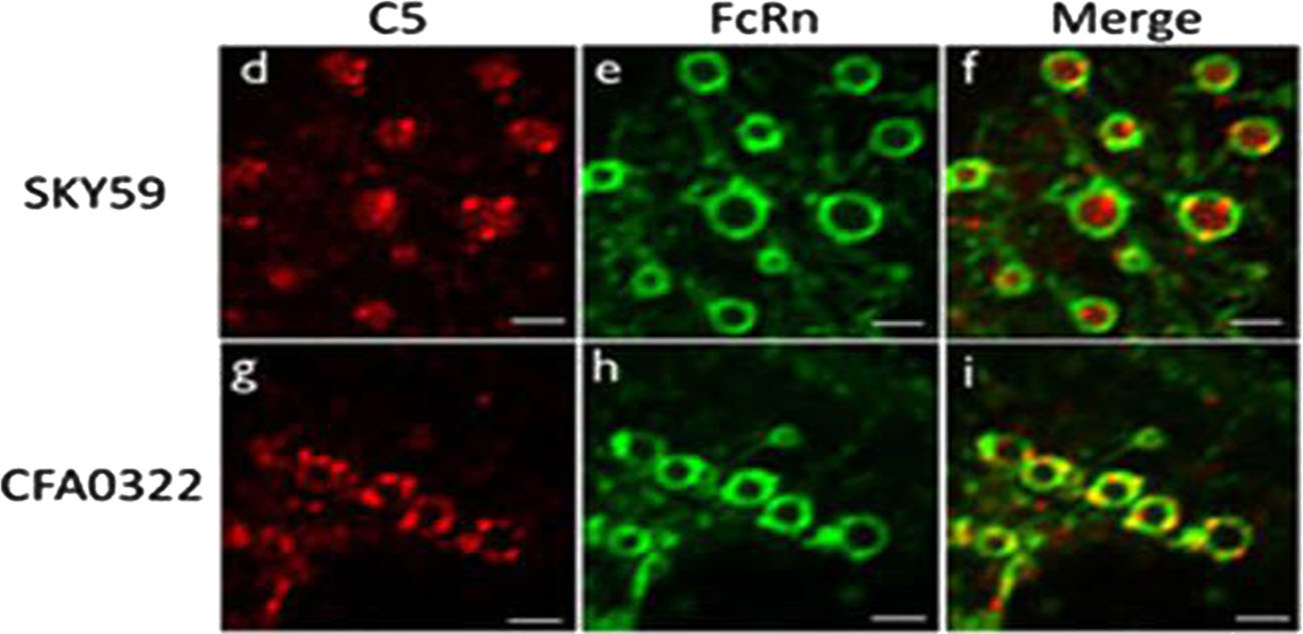
Mode of action of ‘sweeping’ antibodies. Antibodies designed to bind well to antigen and FcRn at pH 7.4 (blood) but to lose binding to antigen at pH 6 can demonstrate a ‘sweeping’ action. This figure, taken from Fukuzawa et al. Sci Rep. 2017 Apr 24;7(1):1080, contrasts the mechanism of C5-binding and C5-release by two monoclonal antibodies: SKY59, a sweeping antibody against C5, and CFA0322, a non-sweeping antibody against C5. These images demonstrate representative images of human C5 localization in endosomal vesicles. MDCK cells expressing human FcRn-EGFP were treated with Alexa 555-labelled C5 and either SKY59 (**d**–**f**) or CFA0322 (**g**–**i**). Red and green represent C5 and FcRn, respectively. C5 was released into the endosomal space in cells treated with SKY59 whilst C5 remained bound on the endosomal membrane in cells treated with (control) CFA0322. Scale bars represent 2 μm. The experiments were repeated twice for representative images. This figure is adapted from the following publication: Fukuzawa T, Sampei Z, Haraya K, Ruike Y, Shida-Kawazoe M, Shimizu Y, et al. (2017). ‘Long lasting neutralization of C5 by SKY59, a novel recycling antibody, is a potential therapy for complement-mediated diseases’. Sci Rep 7:1080. 10.1038/s41598-41017-01087-41597; the original article can be accessed at this URI http://www.nature.com/articles/s41598-017-01087-7 [53]. It is licensed under a Creative Commons Attribution 4.0 International License https://creativecommons.org/licenses/by/4.0/legalcode [53]

Various approached have been developed to modulate drug dose, including engineering of either, or both, the Fc and Fab domains of antibodies. Recycling, or ‘pH switched’, antibodies have been developed to reduce dose [49, 50]. Antibody-based therapeutics usually bind target with high affinity and circulate as a complex until cleared. Typically, the complex is internalised into the endosome compartment of cells where the Fc of the drug binds to FcRn in the slightly acidic environment (pH 6). Binding to FcRn ‘rescues’ the antibody as it is recycled to the cell surface where it is released due to the neutral pH of blood (pH 7.4), at this pH the affinity for Fc and FcRn is lowered. If antigen or target-binding to the antibody idiotype is not impacted at pH 6, then the target recycles along with antibody and the complex is released back to the bloodstream, a process which can result in accumulation of target in blood, sometimes as much as 1000-fold [50, 51]. Recycling antibodies are engineered to take advantage of the acidic environment of the endosome. The antigen-binding domain is modified, often by substituting histidine residues into the CDRs, this introduces pH-dependent binding and weakens affinity for antigen at pH 6 such that the antigen is released in the endosome following internalisation. Endosome/lysosome fusion results in degradation of the soluble antigen and recycling of the antibody bound to FcRn via the sorting endosome back to the cell surface. This releases antibody back to the circulation that is free to bind target once more, hence drug dose is reduced. The first therapeutic antibody to be pH switched was tocilizumab (anti-IL6 receptor), reported in 2010 [49]; however, this strategy has since been employed to generate long-lived anticomplement antibodies. The ‘next-generation eculizumab’ antibody, ALXN1210, is an engineered form of eculizumab which has marginal reduction in affinity for C5 at pH 7.4, but marked reduction at pH 6 [52]. ALXN1210 is currently administered intravenously every other month for patients with PNH (clinical trial identifiers: NCT02946463, NCT03056040). In this case, the recycling technology was applied to an existing agent, and there are a number of other published examples of histidine-based engineering of antibody CDRs; however, this approach poses the risk of impacting binding to target at neutral pH in the circulation. Others have set out to identify antibodies with high potential for pH switching during the drug screening process. Chugai Pharmaceuticals generated monoclonal antibodies from C5-immunised rabbits and screened for those with lower binding affinity at pH 5.8 compared to pH 7.4 and identified a clone with undetectable binding at pH 5.8 and nanomolar affinity (KD) at pH 7.4. This antibody was humanised and further mutations introduced to the variable region to improve affinity, resulting in an antibody (SKY59/RG6107; collaboration with Roche) with subnanomolar affinity (KD 0.152 nM) and a 1000-fold lower binding affinity for C5 at pH 5.8 [53]. Exquisitely designed in vitro studies utilising MDCK (Madin-Darby Canine Kidney Cells) cells expressing FcRn-EGFP and incubated with Alexa 555-labelled C5 and either SKY59 or a control antibody provided compelling evidence for release of C5 from SKY59 within the endosome. The target, C5, could be seen to accumulate within the endosomal space, whereas C5 bound to the control antibody (which did not demonstrate pH dependence of binding) colocalised with FcRn at the membrane (Fig. 4). Crystal structures revealed the epitope of SKY59 on C5 which differs from that of eculizumab and is distal to a polymorphism in C5, R885H, which precludes eculizumab binding to target. In vivo studies in mice and cynomolgus monkeys validate the positive effect of recycling technology on drug PK and clearance of C5; in addition C5 did not accumulate in plasma of monkeys treated with SKY59 [53]. Clinical experience with ALXN1210 validates this antibody engineering approach and we await data from the current phase 1/2 clinical trial of SKY59 in PNH which also utilises subcutaneous injection route rather than intravenous infusion.

Other strategies are used to improve performance of antibody-based drugs such as affinity maturation or mutation within the CDRs to improve affinity for target. Unless complement-dependent cytotoxicity (CDC) or antibody-dependent cellular cytotoxicity (ADCC) are desired modes of action, antibodies which bind cell surface targets are usually modified to eliminate binding of the first complement component, C1q, and to Fcγ receptors (FcγR). These modifications typically involve switching to the IgG4 isotype which lacks these binding interactions, or point mutations within the Fc domain of heavy chain of an IgG1 antibody [54]. However, other Fc engineering strategies can improve therapeutic potency of antibodies, such as mutating the binding site for FcRn to increase the slow rate of non-specific uptake into cells. If this strategy is applied to a pH-dependent antibody, it results in a ‘sweeping’ antibody with enhanced capacity to bind to FcRn at the cell surface and deliver target to lysosome for degradation whilst recycling the antibody to the surface of the cell [55, 56]. A sweeping antibody can bind FcRn at neutral pH on the cell surface, resulting in accelerated uptake and degradation of larger amounts of soluble antigen compared to that achieved with an antibody with recycling properties alone; for sweeping technology to work, pH dependence to release antigen in the endosome is essential [55]. The affinity of antibody for FcRn at neutral pH can impact its rate of clearance; therefore, the balance between accelerated target clearance due to the sweeping action and enhanced antibody clearance must be taken into consideration when considering the engineering strategy. A moderate increase in FcRn affinity may be sufficient to enhance drug potency without impacting clearance [55].

One further strategy for improving antibody PK is emerging but not yet tested in humans. FcyRIIb plays a key role in uptake of immune complexes in the liver and possibly represents a ‘more natural’ route for antigen removal than via the widely expressed FcRn. It is not known whether systemic clearance via FcRn is preferable to liver-mediated clearance via FcyRIIb, but in vivo studies in transgenic mice indicate that engineering the binding site for this receptor in combination with pH switching accelerated clearance of soluble antigen from the circulation [57].

## Targeting neoepitopes at sites of disease

Most complement proteins are present in plasma at high levels; properdin (2 μg/ml), CFD (25 μg/ml) and MASP2 (300 ng/ml) have lower circulating concentrations which may simplify drug delivery, but drug dose may be impacted by turnover; this is certainly the case for CFD. Most complement proteins have a normal concentration which is at least approaching 0.1 g/L and many are much higher (CFB, normal range (n.r.) 0.295–0.4 g/L; C3 n.r. 0.75–1.65 g/L; C4 n.r. 0.14–0.54 g/L; C5 n.r. 0.09–0.172 g/L; C9 n.r. 0.05–0.25 g/L; ranges currently cited by Cardiff and Vale University Health Board Immunology Reference Laboratory). These target concentrations necessitate huge amounts of drug such as that evidenced by eculizumab (anti-C5) described above. Various strategies exist to lower dose and enable subcutaneous administration, including antibody engineering strategies described above, but another approach is to target neoepitopes on complement proteins which appear as the proteins become activated and change conformation. Rather than saturating the system with sufficient drug to take out an entire component of the system, it is possible to develop agents which bind to freshly exposed faces only present on the activated proteins. These agents may be antibodies, and there are abundant reagents with similar binding properties developed for techniques such as immunoassay-based detection of activation products in plasma, or immunohistochemical detection of activated complement deposited in tissues, or they may be proteins which bind activated complement as part of their natural function, such as complement receptors or complement control proteins.

### Antibodies against neoepitopes on complement proteins

Various antibody-based strategies have been developed for targeting complement neoepitopes and several agents are progressing through clinical development. IFX-1 (CaCP29) is a humanised anti-neo antibody which targets C5a but has no impact on MAC formation. Unlike the many other agents currently under development which target at the level of C5, IFX-1 does not bind the native C5 protein but only the activated C5a product; by binding C5a, it prevents engagement of receptors on leukocytes such as neutrophils and T cells and thus prevents the inflammatory cellular response. The agent binds C5a generated via the complement C5 convertase and other enzymes, such as trypsin and thrombin, thus has potential in disease indications, such as aHUS, where C5a may be produced via multiple mechanisms [58]. It is in an advanced stage of clinical development with various phase 2 trials ongoing or completed. The SCIENS phase 2 trial (‘Studying Complement Inhibition in Early, Newly Developing Septic Organ Dysfunction’; NCT02246595) enrolled patients with early organ dysfunction (within 3.5 h of screening); IFX-1 effectively blocked C5a in a dose-dependent manner and showed positive trends in other endpoints such as organ dysfunction score, need for ventilator support and length of stay in intensive care. A phase 2 trial in cardiac surgery was designed to evaluate whether prophylactic treatment with IFX-1 could block the systemic (sterile) inflammatory response often seen following major surgery, particularly the severe and acute systemic inflammation and subsequent organ dysfunction evident following cardiac surgery. The trial (CARDIAC; ‘Studying Complement Inhibition in Complex Cardiac Surgery’; NCT02866825) has completed. A third phase 2 trial in moderate to severe Hidradenitis Suppurativa (HS) has been ongoing (2017; NCT03001622); HS is a common and painful, long-term skin condition that causes abscesses and scarring on the skin. Patients received weekly dosing of IFX-1 for 8 weeks with an expected impact on both neutrophil and T cell function. At the time of writing, data are awaited from these two clinical trials. This agent represents a significant advance in the field as a therapeutic approach with potential to treat both acute and chronic inflammatory disease.

Other drugs which target neoepitopes include a humanised monoclonal antibody, H17 (Elusys Therapeutics), which specifically binds activated C3b and the downstream fragment, iC3b, and blocks the amplification loop [59]. This agent has potential for clinical application in alternative pathway-mediated disease, such as C3G [60]. Genentech employed phage display technology to generate an antibody, S77, which bound C3b but not native C3 [61]. Structural studies of S77 and H17 in complex with C3b indicate that both agents bind C3b within the macroglobulin 6 (MG6)-MG7 region and sterically block interaction with CFB. The central complement component, C3, turns over at a high rate in plasma due to tickover and it is not yet clear how this impacts dosing of such agents; recent clinical development is not reported for either agent.

Several other agents have been reported with preferential binding to activated complement, but as yet little data are available. An anti-neo antibody specific for activated C1s, TNT020, is reported in early-stage development by True North Therapeutics. This agent follows in the wake of TNT009, a monoclonal antibody which is in clinical development for various complement-mediated disorders including bullous pemphigoid (BP) and cold agglutinin disease.

### Targeted delivery to neoepitopes

An alternative strategy to anti-neo antibodies is to ‘home’ agents to sites of complement attack using engineered fragments of proteins or antibodies with affinity only to activated complement—this includes binding domains of complement receptors and regulators with activities as described above. One agent, TT30, is a chimeric molecule comprising the iC3b/C3dg-binding domain of complement receptor 2 (CR2, CD21) and the functional domains of CFH, the resultant molecule ‘homes’ to sites of complement activation via the CR2 domain and delivers therapy due to decay accelerating and cofactor activities residing within the CFH moiety [62]. This agent demonstrated marked therapeutic benefit in various animal models and was initially developed by Taligen Therapeutics, later being acquired by Alexion Pharmaceuticals and tested in man in PNH (phase 1) [63]. The agent was administered by single-dose intravenous infusion or separately by subcutaneous injection; there were no dose-related safety risks and a transient pharmacologically relevant inhibition of complement was evident in patients at the higher doses. Further development of this agent has not yet been reported. One other “homing” agent to make it to clinical development is Mirococept (APT070). This agent comprises functional domains from CR1 tagged to a lipid ‘tail’ which facilitates binding to membranes [64]. It is currently in phase 2 trial for delayed graft function (Mirococept (APT070) for preventing ischaemia reperfusion injury in the kidney allograft; EMPIRIKAL); the cytotopic property of Mirococept endowed by the lipid tail enables an innovative ex vivo delivery strategy whereby the agent is localised within the microvasculature of the donated organ by perfusion prior to transplant. This approach limits systemic effects whilst maximising localised inhibition of complement. The trial should read out towards the end of 2017 [65].

Other agents in preclinical phase of development include ‘mini-fH’, an engineered form of CFH comprising the amino-terminal functional domains, which have decay accelerating and cofactor activity, fused to the carboxy-terminal domain, the resulting molecule binds avidly to self-surfaces undergoing complement attack [66]. Various forms of this engineered ‘mini’ form of CFH have been tested in vitro and in animal models and appear to be more effective on a molar basis than the native CFH at protecting self-surfaces; one form of mini-fH, AMY-201, is in preclinical development by Amyndas Pharmaceuticals [67]. These agents all deliver inhibition of the activation pathways of complement; however, one agent has been developed which inhibits at the terminal stage and prevents MAC formation; this has been tested in mice. In this study, the iC3b-binding domain of complement receptor of the Ig superfamily (CRIg) was fused to the MAC-inhibiting domain of the MAC inhibitor, CD59; the molecule was dimerised by using recombinant DNA technology to insert the hinge region of murine IgG2a (generating CD59-2a-CRIg) and was shown to bind avidly to surfaces attacked by complement [68]. CRIg binds to the C3c domain of activated C3 and thus binds iC3b, but not C3dg; homing to iC3b should focus the molecule to freshly activated complement, rather than ‘old’ hot spots of complement activation which are likely decorated in the end-stage degradation fragment, C3dg, rather than iC3b [69]. This agent was administered systemically (intravenously) 30 min post-injury in a murine model of traumatic brain injury (TBI) and successfully localised to the damaged tissue. It decreased clinical severity score and prevented MAC formation, axonal damage, cell stress and microglial activation. These data illustrate the therapeutic potential of homing agents but there are unanswered questions which should be addressed for further optimisation of such molecules in man. What is the appropriate homing target in man—a species that lacks the widespread rodent complement regulator Crry (complement receptor 1-related protein/gene y) which supports cleavage of iC3b to C3dg? The cofactor enabling factor I to produce C3dg from iC3b in man (CR1) has a limited distribution—does this mean that the blood and tissue distribution of iC3b and C3dg differ across species? What impact does the fluid phase tickover of the alternative pathway have on dose—is there a massive fluid phase sump of C3 fragments that must be overcome to home effectively? Can these agents provide therapy in diseases which have an element of fluid phase dysregulation? Likely the answers to these questions will differ according to the disease indication and pathogenic mechanism but will need to be addressed to advance the field to clinical development.

One approach which may circumvent this issue is to home therapeutic molecules to non-complement antigens which appear on diseased or damaged tissue [70]. One strategy that has shown promise in preclinical models is to deliver to antigens exposed due to tissue trauma or stress. These damage-associated molecular patterns (DAMPs) usually trigger an innate immune response and are associated with damage resulting from a number of autoimmune and inflammatory diseases and injury arising from conditions such as ischemia-reperfusion. These antigens include ligands which are present on early and late apoptotic/necrotic cells and are bound by natural antibodies whose role is to ‘sense’ danger. A number of monoclonal antibodies have been generated which recognise these antigens on stressed cells in mice and they demonstrate specificity for modified annexin-4 (ANX4) and modified phospholipids [71]. A scFv developed from the anti-ANX4 antibody has been further developed as a chimeric agent with a complement inhibitor and has shown to home to the relevant site and protect against post-transplantation cardiac reperfusion injury [72]. The possibility for translational to man is supported by the presence of anti-ANX4 antibodies in the plasma of a number of normal humans; in general, stress-related antigens conserve well across species.

Activated complement products are at higher concentration at sites of disease, with activation fragments often covalently tagged to the cell surface. Generation of therapeutic agents which specifically target or deliver to the surface-associated antigens not only circumvents the challenge of dosing for a high target concentration but also opens up the possibility of localised, site-targeted therapy. The ideal ‘homing’ or ‘targeting’ agent should be capable of being administered systemically, it should home to target specifically at disease site without encountering any depot of ligand on the way, and once delivered it should be retained at disease site to provide lasting therapy. The complement-regulating moiety should have a ‘recycling’ activity whereby it engages with a specific conformation of target (for example, activated convertase, C3bBb) to mediate a change such as decay of a complex, or irreversible structural modification (such as cleavage by CFI). It should then release and rebind to fresh active target, thus preserving its ability to control complement at that site. The exception to this might be when limited short-term therapy is all that is required, such as that provided in TBI by the CD59-2a-CRIg agent described above. This strategy of localised delivery has the distinct advantage of minimising systemic inhibition and long-term risk of infection. It is logical that homing agents should have these properties, but there are a number of unknowns to be explored such as the affinity of the homing arm for target—is it preferable to deliver an agent with high avidity for binding the target or with low affinity and ability to release and rebind rapidly at the surface? The possibility of addressing this question is opened up by availability of monomeric and multimeric forms of homing agents and also by use of recombinant antibody for delivery, not only can antibody-based agents bind with multiple arms but also mutation within the CDRs can generate agents with lower or higher specificity for target antigen enabling in vivo experimentation to answer this question.

## Structure-based design for orally bioavailable molecules

Intricate structural information is now abundant in the complement field; this has not only provided critical insight into the mechanism of action of existing drugs such as the C3 inhibitor, AMY-101 (compstatin derivative) [39, 73], but it has also facilitated structure-based design of new drugs. Various small molecules (SM) which are orally bioavailable are now under development for use in man to inhibit complement; this delivery strategy represents an exciting leap forward for the anticomplement field and has been enabled due to the availability of high resolution crystal structures for individual complement proteins, such as CFB and CFD, and complement complexes, such as the trimolecular complex of C3b, CFB and CFD [20]. Oral bioavailability may circumvent current issues with drug delivery. Both Achillion Pharmaceuticals and Novartis have developed SM inhibitors of CFD. The Novartis molecule was developed using a two-pronged methodology combining structure-based design with fragment-based screening [26]. Identification of potential moieties that could dock into the CFD active site was steered by prior knowledge of fragment binding to the related serine protease, kallikrein-7. This ‘target-hopping’ approach guided in silico docking of compounds to the crystal structure of CFD and identified those molecules suitable for synthesis and subsequent testing for CFD inhibitory activity in vitro. A weakly inhibiting compound was identified which was shown to bind to CFD using [1 H, 15 N]-HSQC NMR spectroscopy. Further in silico screening of a fragment (< 300 Da) library identified a set of fragments docking into the active site of CFD and one of these was shown to interact in vitro using ligand-observation NMR. Guided by the molecular structure of both these hits, a small molecule was designed and optimised with improved affinity and selectivity and inhibitory activity in vivo in an LPS-induced systemic and ocular complement activation model in C57Bl/6 mice expressing human CFD [26]. Novartis have also developed orally bioavailable SM inhibitors of CFB; clinical progression of these two molecules is anticipated. The Achillion CFD inhibitor, ACH-4471, is also orally bioavailable and is progressing through clinical development with announcement of phase 2 studies expected shortly [25, 48]. The screening procedure for the Achillion molecule is not published but was likely powered by structure-guided design, a process central to the company’s drug discovery process. This exciting era where in depth structural data can augment drug discovery to deliver highly specific and potent molecules is likely to change the landscape of complement drug discovery in the coming years. It is intriguing to speculate that the recently available structures of the complement inhibitors in complex with their ligands, will also guide development of SM capable of controlling the activation complexes of the complement cascade [74, 75].

The examples above illustrate how molecular structure of a target can guide design of new small molecule entities; however, prior knowledge of drug binding to target can also be used to discover new molecular entities of similar or different modality. RaPharma utilised their mRNA *Extreme Diversity*™ display platform to screen for macrocyclic peptides which bound to C5. Using this approach, the company has developed a drug in clinical development, RA101495, which blocks terminal pathway. This molecule binds both C5 and C5b and thus blocks MAC formation triggered by both C5 convertase and by non-complement serine proteases, such as thrombin and trypsin. Its molecular properties of mass, potency and bioavailability enable once-daily subcutaneous dosing; it is currently in phase 2 PNH trials at a dose of 0.1–0.3 mg/kg (NCT03030183, NCT03078582). The crystal structure of RA101495 peptide bound to a functionally relevant domain of C5 provided sufficient in depth understanding of the inhibitory mechanism to enable structure-guided optimisation of SM drugs [76]. These molecules display a differentiated mechanism of action compared to anti-C5 monoclonal antibody therapies and the lead series is reported to be orally bioavailable; data from in vitro functional assays indicate activity in whole serum.

## Targeting at the genetic level to overcome high protein concentration

The strategies described above have evolved to overcome the challenges in complement drug discovery such as target concentration, target turnover and infection risk. These approaches which include antibody engineering, targeting of neoepitopes and development of orally bioavailable molecules are now being validated in the clinic and represent a huge step forward in complement therapeutics. However, an alternative line of attack is to modify protein biosynthesis by targeting at the genetic level—this might be to prevent expression of an activating protein or increase expression of an inhibitory protein. Ionis Pharmaceuticals are leaders in antisense technology and have developed an array of antisense oligonucleotides (ASO) targeting translation of complement proteins, including CFB; ASO-targeting of CFB reduced levels of circulating CFB (to < 20% normal levels) and improved survival, proteinuria and renal pathology in murine lupus nephritis [77]. The company also reported that systemic administration to mice and monkeys of CFB ASO impacted levels of CFB in tissues other than the liver/circulation, such as the eye, holding promise for therapy of organ-specific disease [78]. The Ionis asset which targets CFB, IONIS-FB-LRx, is currently in clinical development, although phase 1 data are not yet reported. Alnylam Pharmaceuticals have developed an RNAi therapeutics platform for the delivery of siRNAs to the liver using trivalent GalNAc conjugates and have described an asset, ALN-CC5, which silences hepatocyte expression of C5 following subcutaneous injection. This agent effectively suppressed liver biosynthesis of C5 in man with a single dose of 600 mg knocking down C5 levels in plasma by 97% up to day 98 post-treatment; maximum knockdown relative to baseline was 99% [79]. It is likely that complete knockdown was unattainable due to extrahepatic biosynthesis of C5. The first indication in which ALN-CC5 has been tested is PNH (phase 1/2, NCT02352493), a challenging disease to treat as experience with eculizumab has taught us that total knockdown of C5 is required to completely ablate erythrocyte lysis; however, combination therapy with eculizumab has permitted exploration of potential for reducing dose and frequency of eculizumab in PNH patients. Whether ALN-CC5 continues clinical development for PNH remains to be seen, but it has promise for other indications where complete knockdown is not essential.

There remains untapped potential for modulating complement at the genetic level using cell- or gene-targeting strategies [80, 81]. These approaches may enable localised and long-term expression of additional complement control proteins to provide therapy at sites of disease. This represents an alternative strategy for localised therapy and for sparing of systemic complement to minimise risk and side effects. Increasing the level of control provided naturally by the body to prevent complement-mediated damage is a sensible strategy. It is notable that man has evolved highly effective mechanisms to successfully control complement and maintain health even in the face of excessive inflammation and infection; the intricate mechanisms of decay acceleration and cofactor activities are starting to be unravelled and we may do well to take heed of lessons of nature and think about how we might modulate complement (rather than block) to bring about a healthy balance between activation and control that minimises risk and secondary complications of therapy.

## Concluding remarks

Clinical validation with a variety of agents either approved or in late-stage clinical development has confirmed that anticomplement therapy can have astounding impact on improving quality of, and preserving, life in a growing number of diseases. There are limitations to many of the drugs under development but those shortcomings are driving an evolution in complement drug discovery which will change the landscape for the better. Within the next decade, clinicians will have at their disposal a toolbox of anticomplement drugs which can be tailored to the right disease indication to bring about profound clinical benefit, can be administered at home by the patients themselves and are available at a cost which opens access to common diseases and patients worldwide.
